# P04.39. Leadership and education program for students in integrative medicine: LEAPS into IM

**DOI:** 10.1186/1472-6882-12-S1-P309

**Published:** 2012-06-12

**Authors:** M Guerrera, W Kohatsu, H Roca, J Polli, N Kaveh, B Berman-Brady

**Affiliations:** 1 University of Connecticut School of Medicine, Hartford, USA; 2 Santa Rosa Family Medicine Residency, Santa Rosa, USA; 3 Yale School of Medicine, New Haven, USA; 4 Consortium of Academic Health Centers for Integrative Medicine , Minneapolis, USA; 5 American Medical Student Association, Reston, USA; 6 A.T. Still University School of Osteopathic Medicine , Mesa, USA

## Purpose

To present preliminary data from LEAPS into IM, the Leadership and Education Program for Students in Integrative Medicine, an innovative program designed to foster the development of the next generation of medical student leaders in Integrative Medicine (IM). Medical students selected from across North America engaged in hands-on and didactic IM education, leadership skills, self-care, and community building curricula with leading experts serving as teachers/mentors. Our goal was to evaluate LEAPS impact on several IM related behaviors in participants immediately following and several months after the program.

## Methods

Allopathic and osteopathic medical students who completed the LEAPS into IM program in 2010 (N=20) and 2011 (N=30) were surveyed at the conclusion of the week-long program and quarterly thereafter via an anonymous IRB approved survey. Questions explored participants’ utilization of Integrative Medicine resources in self-care and IM referral for family, friends, and patients. Satisfaction with medicine was also measured. Additional questions included other exposure to integrative medicine educational material over time.

## Results

Students exhibited increased utilization of yoga, group activities, exercise, and healthy nutrition a year after conclusion of the program. A significant drop in group support activity at 6 months correlated with a drop off in return of follow-up surveys and an apparent reduction in satisfaction with standard medical education. Comparison across years showed similar results.

## Conclusion

The LEAPS into IM Program appears to be effective in enhancing student utilization of select Integrative Medicine resources and referrals. A significant correlation exists between continued engagement with Integrative Medicine and the support given by groups of like-minded individuals.

